# Health Disparities in Cardiac Critical Care: Understanding Inequities, Barriers, and Pathways Toward Equitable Access and Enhanced Outcomes

**DOI:** 10.7759/cureus.64151

**Published:** 2024-07-09

**Authors:** Mohamed R Abouzid, Ibrahim Kamel, Ibrahim Elkhawas, Amro Rezk, Sadaf Esteghamati, Chima C Nwaukwa

**Affiliations:** 1 Internal Medicine, Baptist Hospitals of Southeast Texas, Beaumont, USA; 2 Internal Medicine, Tufts Medical Center, Boston, USA; 3 Internal Medicine, Steward Carney Hospital, Boston, USA; 4 Internal Medicine, Steward Carney, Dorchester, USA; 5 Internal Medicine, Kasr Al Ainy Medical School, Cairo, EGY; 6 Internal Medicine, University of La Verne, La Verne, USA; 7 Cardiology, Baptist Hospitals of Southeast Texas, Beaumont, USA

**Keywords:** ethnic disparities, racial disparities, geographic barriers, health insurance, socioeconomic factors, interventions, outcomes, access, cardiac critical care, health disparities

## Abstract

Health disparities in cardiac critical care continue to pose significant challenges in achieving equitable access and outcomes for diverse populations. This literature review examines the disparities in access to and outcomes of cardiac critical care interventions across different populations, identifies barriers contributing to these disparities, and explores strategies to address them. A literature review was conducted by searching electronic databases for relevant articles published between January 2000 and May 2023. Studies focusing on health disparities in cardiac critical care, access to interventions, outcomes, and equity were included. Data were extracted and synthesized using a narrative approach. Disparities in access to cardiac critical care interventions were identified, including socioeconomic factors, lack of health insurance, geographic barriers, racial and ethnic disparities, language and cultural barriers, limited health literacy, and lack of awareness and education. These barriers led to delayed diagnoses, suboptimal utilization of interventions, and limited access to specialized cardiac care. Disparities in outcomes were also observed, with certain populations experiencing worse clinical outcomes and higher morbidity and mortality rates. This review emphasizes the existence of disparities in cardiac critical care and emphasizes the necessity for interventions to address these disparities. Specific strategies should concentrate on enhancing healthcare access, diminishing financial obstacles, expanding health insurance coverage, fostering patient-centered approaches, and harnessing telemedicine and technology. Collaborative efforts among policymakers, healthcare providers, researchers, and patient advocates are vital to advocate for policy changes and implement evidence-based interventions that foster equitable care. Future research should prioritize longitudinal studies, implementation science, patient engagement, global perspectives, and rigorous evaluation of intervention strategies to advance our knowledge and guide endeavors in reducing health disparities in cardiac critical care.

## Introduction and background

Health disparities in cardiac critical care represent a significant challenge in achieving equitable care across different populations. Cardiac critical care interventions, including cardiac surgery, percutaneous coronary intervention, and cardiac arrest resuscitation, are essential for improving patient outcomes and reducing mortality rates. However, certain populations face barriers that limit their access to quality care, resulting in disparities in health outcomes and exacerbating existing health inequities [1,2].

Health disparities are defined as unjust and avoidable differences in health outcomes between populations [1]. These disparities are influenced by a complex interplay of social, economic, cultural, and healthcare system factors. In the context of cardiac critical care, disparities in access and outcomes can have profound implications for patient survival, recovery, and quality of life [3,4].

Understanding and addressing health disparities in cardiac critical care are crucial to promoting equitable care and reducing avoidable morbidity and mortality. By examining the patterns of disparities and identifying the barriers that contribute to these inequalities, healthcare systems and policymakers can develop targeted interventions, implement policy changes, and allocate resources effectively to improve access and outcomes for all patients [1].

Access disparities in cardiac critical care occur across multiple dimensions. Socioeconomic factors, such as income, education level, and health insurance status, play a significant role in determining access to cardiac critical care interventions. Financial constraints, lack of insurance coverage, and limited healthcare resources can hinder timely presentation, referral patterns, and transportation to appropriate care facilities. Individuals from lower socioeconomic backgrounds often face challenges in accessing specialized cardiac care, leading to delayed diagnosis, suboptimal management, and poorer outcomes [5,6].

Racial and ethnic disparities also contribute to inequities in access to cardiac critical care interventions. Disparities have been observed among racial and ethnic minority populations, including African Americans, Hispanics, and Indigenous communities [7]. These disparities may arise due to systemic factors, including implicit biases, discrimination, and structural inequities in healthcare delivery. Patients from these populations may experience lower rates of cardiac procedures, longer wait times, and underutilization of guideline-directed therapies, leading to suboptimal outcomes [2].

Geographic disparities pose additional challenges in accessing cardiac critical care. Patients residing in rural areas often encounter limited availability of specialized cardiac care centers, longer travel distances, and a shortage of healthcare professionals. These geographical barriers contribute to delays in receiving timely interventions and accessing the necessary expertise, resulting in disparities in access and outcomes [1,8].

The impact of health disparities extends beyond access to cardiac critical care interventions and encompasses disparities in health outcomes. Disadvantaged populations, including those with lower socioeconomic status and racial and ethnic minorities, experience higher mortality rates, poorer long-term outcomes, and increased rates of complications following cardiac critical care interventions [9]. These disparities are influenced by a combination of factors, including delayed access to care, limited healthcare resources, lower health literacy, and social determinants of health [8].

Addressing health disparities in cardiac critical care requires a comprehensive approach that encompasses targeted interventions, policy changes, and improvements in healthcare delivery. By identifying the barriers that contribute to disparities and understanding the underlying causes, healthcare systems can develop strategies to promote equitable care, improve access, and enhance outcomes for all patients [10].

This literature review aims to provide a comprehensive overview of the existing research on health disparities in cardiac critical care. By synthesizing the findings from relevant studies, this review seeks to contribute to the understanding of disparities in access and outcomes across different populations. Furthermore, it aims to identify gaps in the current knowledge and highlight potential avenues for future interventions, policy changes, and research in the pursuit of equitable care in cardiac critical care settings.

## Review

Methodology

Study Objective

The objective of this review is to examine the health disparities and inequities in access to and outcomes of cardiac critical care interventions across different populations and identify barriers and strategies to address these disparities.

Study Design

This review follows a literature review approach. A comprehensive search of electronic databases, including PubMed, Embase, and Scopus, was conducted to identify relevant articles published between January 2000 and August 2023. The search strategy used a combination of keywords related to health disparities, cardiac critical care, access, outcomes, and equity.

Inclusion and Exclusion Criteria

Studies were included if they focused on health disparities in cardiac critical care, examined access to and outcomes of cardiac interventions, and provided data on different populations or subgroups. Only peer-reviewed articles written in the English language were included. Studies that primarily focused on pediatric populations or non-cardiac critical care were excluded.

Data Extraction

Two independent reviewers screened the titles and abstracts of the identified articles to assess their relevance to the research objective. Full-text articles of potentially relevant studies were then reviewed to determine their eligibility for inclusion.

Data Synthesis

The extracted data were synthesized using a narrative approach. Key findings related to health disparities in access to cardiac critical care interventions and disparities in outcomes were summarized. The barriers contributing to these disparities were identified and grouped into thematic categories. Strategies and interventions to address disparities were also synthesized and categorized.

Limitations

The limitations of the included studies and the review itself were considered and reported. These limitations included factors such as publication bias, heterogeneity in study designs and populations, the potential for bias in data extraction, and the generalizability of the findings.

Ethical Considerations

This review only utilized publicly available data from previously published studies. Therefore, ethical approval was not required.

The methodology employed in this review aimed to ensure a comprehensive and systematic approach to identify relevant studies, extract data, synthesize findings, and assess the quality of the included studies. By adhering to established guidelines, the review aimed to minimize bias and provide a robust overview of the health disparities in cardiac critical care.

Discussion

Health disparities in cardiac critical care represent a significant challenge in achieving equitable care across different populations. This literature review has highlighted several key findings related to the access and outcomes of cardiac critical care interventions, as well as identified barriers and potential strategies to address health disparities.

Disparities in Access to Cardiac Critical Care

Socioeconomic disparities: Individuals with lower socioeconomic status often face barriers to accessing cardiac critical care interventions. Factors such as income, education level, and insurance coverage can significantly impact access. Financial constraints and lack of adequate insurance coverage can result in delayed diagnosis, limited treatment options, and reduced access to necessary healthcare services [5].

Racial and ethnic disparities: Disparities in access to cardiac critical care interventions have been observed among different racial and ethnic groups. Minority populations, including African Americans, Hispanics, Indigenous communities, and other racial/ethnic minorities, may experience lower rates of cardiac procedures, longer wait times, and reduced access to specialized cardiac care facilities. Structural inequities, systemic biases, and limited healthcare resources in underserved communities contribute to these disparities [11-13].

Geographic disparities: Access to cardiac critical care can vary based on geographic location. Individuals residing in rural or remote areas often face challenges in accessing specialized cardiac care services. Limited availability of cardiac care centers, long travel distances, and a lack of transportation options can result in delayed or suboptimal access to critical interventions [8].

Health insurance disparities: Disparities in insurance coverage can significantly impact access to cardiac critical care. Individuals without health insurance or with limited coverage may face financial barriers that hinder their ability to access timely cardiac interventions. Lack of insurance can lead to delayed diagnosis, limited treatment options, and decreased utilization of necessary healthcare services [14].

Language and cultural barriers: Language and cultural barriers can contribute to disparities in accessing cardiac critical care, particularly among individuals with limited English proficiency or from non-English-speaking backgrounds. Communication challenges can impact understanding, patient-provider interactions, and the ability to navigate the healthcare system effectively [15].

Disparities in Outcomes of Cardiac Critical Care

Mortality rates: Disparities in mortality rates have been observed among different populations receiving cardiac critical care interventions. Certain groups, such as racial and ethnic minorities and individuals of lower socioeconomic status, may experience higher mortality rates following cardiac procedures compared to their counterparts. These disparities may be influenced by factors such as delayed diagnosis, limited access to specialized care, and variations in treatment quality and follow-up care [14].

Complication rates: Disparities exist in the occurrence of complications following cardiac critical care interventions. Some populations may be at a higher risk of experiencing complications, such as surgical site infections, postoperative complications, or adverse events related to medication management. Factors contributing to these disparities include variations in preoperative management, comorbidities, and access to quality follow-up care [1,14].

Functional status and quality of life: Disparities in functional status and quality of life outcomes can be observed among individuals who have undergone cardiac critical care interventions. Some populations, particularly those facing socioeconomic or racial/ethnic disparities, may experience poorer functional outcomes and lower quality of life post-intervention. Factors contributing to these disparities include disparities in access to cardiac rehabilitation programs, postoperative care, and social support systems [16].

Health-related readmissions: Disparities in rates of hospital readmissions following cardiac critical care interventions have been reported. Certain populations, including those with lower socioeconomic status or limited access to post-discharge care, may have higher rates of readmissions for cardiac-related issues. Factors such as inadequate follow-up care, medication adherence, and social determinants of health can contribute to these disparities [14].

Long-term survival and disease management: Disparities can exist in long-term survival rates and disease management among populations who have received cardiac critical care interventions. Variations in access to preventive care, medication adherence, and ongoing disease management may contribute to differences in long-term outcomes. Socioeconomic factors, race/ethnicity, and health literacy can play a role in these disparities [16].

Disparities in outcomes among different populations receiving cardiac critical care interventions are evident. Marginalized and underserved groups experience higher mortality rates, poorer long-term outcomes, and increased complication rates. These disparities may be multifactorial, influenced by underlying comorbidities, delayed access to care, and social determinants of health [5,17]. Efforts to improve outcomes should focus on comprehensive care coordination, early identification of high-risk individuals, and tailored interventions addressing social determinants of health [6].

Barriers Contributing to Health Disparities

Socioeconomic factors such as financial constraints, lack of health insurance, and limited access to transportation hinder access to cardiac critical care. Additionally, cultural and language barriers, including language differences, cultural variations, and inadequate cultural competence among healthcare providers, can impede effective communication and understanding, thereby affecting access and outcomes. Healthcare system factors also play a significant role, with fragmented healthcare systems, lack of coordination, and limited availability of specialized cardiac care centers contributing to disparities in access and outcomes [3,6,9,17].

*Strategies to Address Health Disparities* *[18-22]*

Policy-level interventions: Implementing policy changes at the national, regional, or institutional levels can help address health disparities in cardiac critical care. These interventions may include the below.

Expanding health insurance coverage: Ensuring adequate health insurance coverage, including Medicaid expansion or public insurance programs, can reduce financial barriers to accessing cardiac critical care interventions.

Reimbursement mechanisms: Implementing reimbursement mechanisms that incentivize quality care, such as pay-for-performance models, can encourage healthcare providers to deliver equitable care to all patients and improve outcomes.

Health system reforms: Implementing reforms in healthcare systems to promote equity, such as reducing wait times, improving coordination of care, and enhancing access to specialized cardiac care services in underserved areas.

Culturally tailored interventions: Developing interventions that consider cultural beliefs, practices, and language preferences can improve access to and outcomes of cardiac critical care interventions. Some strategies include the below.

Cultural competence training: Providing healthcare professionals with training to enhance their cultural competence and understanding of diverse populations’ beliefs, values, and healthcare-seeking behaviors.

Language services: Ensuring the availability of interpreters and translation services to facilitate effective communication between healthcare providers and patients with limited English proficiency.

Patient-centered approaches: Adopting patient-centered care models that actively involve patients in decision-making processes, respect their values and preferences, and tailor treatment plans to their individual needs.

Community engagement and education: Engaging with communities and providing education about cardiac health, prevention, and the importance of timely intervention can help reduce disparities. Strategies include the following: (1) Community health fairs and outreach programs: organizing health fairs, mobile clinics, or community outreach programs to provide cardiac health screenings, education, and referrals to underserved populations. (2) Health literacy programs: Developing and implementing programs to improve health literacy, providing information in accessible formats, and empowering individuals to make informed decisions about their cardiac health.

Healthcare workforce diversity: Promoting diversity and representation within the healthcare workforce, particularly among cardiac critical care providers, can help address disparities. Strategies include the following: (1) Recruitment and retention programs: implementing programs to recruit and retain healthcare professionals from underrepresented backgrounds in cardiac critical care specialties. (2) Cultural sensitivity training: providing training to healthcare providers to enhance their understanding of cultural norms, implicit biases, and effective communication strategies when caring for diverse patient populations.

Telemedicine and telecardiology: Utilizing telemedicine and telecardiology technologies can help overcome geographic barriers and improve access to specialized cardiac care, particularly in rural or underserved areas.

By considering the implications of this review, stakeholders in healthcare, including policymakers, healthcare providers, researchers, and patient advocates, can work toward reducing health disparities in cardiac critical care and promoting more equitable healthcare delivery and outcomes.

Limitations

This literature review has some limitations. First, the included studies varied in design, populations studied, and measurement tools, which may limit direct comparisons and generalizability. Second, the review focused on published studies from 2000 until May 2023. Third, the included studies might have employed different study designs, methodologies, and sample characteristics, leading to heterogeneity in the data. This heterogeneity can introduce challenges in synthesizing the results and drawing definitive conclusions. Lastly, this review relies on existing studies and data, which might have inherent limitations in study design, data quality, or representativeness. The quality and reliability of the included studies can vary, and the review is dependent on the accuracy and completeness of the original data sources. Acknowledging these limitations is crucial for understanding the scope and implications of the review’s findings and for guiding future research in addressing health disparities in cardiac critical care.

Future considerations for research on health disparities in cardiac critical care

Longitudinal Studies

Future research should incorporate longitudinal study designs to better understand the long-term effects of health disparities in cardiac critical care. By tracking patients over an extended period, researchers can assess the persistence of disparities, evaluate the effectiveness of interventions, and identify factors that contribute to disparities in outcomes over time.

Intersectionality

Future studies should consider the intersectionality of various social identities, such as race, gender, socioeconomic status, and age, to gain a more nuanced understanding of health disparities in cardiac critical care. Examining how multiple dimensions of identity intersect and interact can provide valuable insights into the unique challenges faced by individuals belonging to multiple marginalized groups.

Implementation Science

Future research should focus on implementation science to understand how to effectively translate evidence-based interventions into practice and address barriers to their implementation. By studying the implementation process, researchers can identify strategies that promote the adoption and sustainability of interventions aimed at reducing health disparities in cardiac critical care.

Patient Engagement

Future research should emphasize patient engagement and incorporate patient perspectives in study design, intervention development, and policy-making processes. Engaging patients as active partners can help ensure that interventions and policies align with their needs, preferences, and values, ultimately leading to more patient-centered and equitable care.

Health Information Technology

Further exploration of the role of health information technology in addressing health disparities in cardiac critical care is warranted. Leveraging electronic health records, data analytics, and decision support systems can facilitate personalized care, improve care coordination, and reduce disparities in access to and outcomes of cardiac interventions.

Global Perspectives

While this review primarily focuses on health disparities in cardiac critical care in specific regions, future research should also consider a global perspective. Examining health disparities and disparities in access to cardiac critical care interventions in diverse healthcare systems and cultural contexts can provide insights into effective strategies and policies to promote equitable care worldwide.

Evaluation of Intervention Strategies

Future studies should prioritize rigorous evaluation of intervention strategies aimed at reducing health disparities in cardiac critical care. By assessing the effectiveness, cost-effectiveness, and scalability of interventions, researchers can provide evidence to guide policy decisions and resource allocation to promote equitable care.

Considering these future considerations will help advance the understanding of health disparities in cardiac critical care and guide the development and implementation of interventions that promote equitable access and improve outcomes for all patient populations.

## Conclusions

This literature review underscores the presence of health disparities in cardiac critical care, highlighting disparities in both access to and outcomes of interventions. Barriers such as socioeconomic factors, lack of health insurance, geographic limitations, racial and ethnic disparities, and limited health literacy contribute to inequitable access to specialized cardiac care. These disparities are associated with worse clinical outcomes and higher morbidity and mortality rates among certain populations. Strategies to address these disparities include improving access to care, reducing financial barriers, promoting patient-centered approaches, enhancing cultural competence among healthcare providers, and leveraging telemedicine and technology.

Collaboration among policymakers, healthcare providers, researchers, and patient advocates is crucial to advocate for policy changes and implement evidence-based interventions that promote equitable care and improve outcomes for all individuals affected by cardiac diseases. Future research should focus on longitudinal studies, intersectionality, implementation science, patient engagement, global perspectives, and rigorous evaluation of intervention strategies to advance our understanding and guide efforts in reducing health disparities in cardiac critical care.
